# Diagnostic accuracy of Fever-PAIN and Centor criteria for bacterial throat infection in adults with sore throat: a secondary analysis of a randomised controlled trial

**DOI:** 10.3399/BJGPO.2021.0122

**Published:** 2021-11-24

**Authors:** Anna Seeley, Thomas Fanshawe, Merryn Voysey, Alastair Hay, Michael Moore, Gail Hayward

**Affiliations:** 1 Nuffield Department of Primary Care Health Sciences, University of Oxford, Oxford, UK; 2 Oxford Vaccine Group, Department of Paediatrics, University of Oxford, Oxford, UK; 3 Centre of Academic Primary Care, Bristol Medical School, University of Bristol, Bristol, UK; 4 Primary Care and Population Sciences Unit, University of Southampton, Aldermoor Health Centre, Aldermoor Close, Southampton, UK

**Keywords:** diagnosis, minor illness, respiratory illness, pharyngitis, decision making, general practice, primary healthcare

## Abstract

**Background:**

Sore throat is a common and self-limiting condition. There remains ambiguity in stratifying patients to immediate, delayed, or no antibiotic prescriptions. The National Institute for Health and Care Excellence (NICE) recommends two clinical prediction rules (CPRs), FeverPAIN and Centor, to guide decision making.

**Aim:**

To describe the diagnostic accuracy of CPRs in identifying streptococcal throat infections.

**Design & setting:**

Adults presenting to UK primary care with sore throat, who did not require immediate antibiotics.

**Method:**

As part of the Treatment Options without Antibiotics for Sore Throat (TOAST) trial, 565 participants, aged ≥18 years, were recruited on day of presentation to general practice. Physicians could opt to give delayed prescriptions. CPR scores were not part of the trial protocol but were calculated post hoc from baseline assessments. Diagnostic accuracy was calculated by comparing scores with throat swab cultures.

**Results:**

It was found that 81/502 (16.1%) patients had group A, C, or G streptococcus cultured on throat swab. Overall diagnostic accuracy of both CPRs was poor: area under receiver operating characteristics (ROC) curve 0.62 for Centor; and 0.59 for FeverPAIN. Post-test probability of a positive or negative test was 27.3% (95% confidence interval [CI] = 6.0% to 61.0%) and 84.1% (95% CI = 80.6% to 87.2%) for FeverPAIN ≥4; versus 25.7% (95% CI = 16.2% to 37.2%) and 85.5% (95% CI = 81.8% to 88.7%) for Centor ≥3. Higher CPR scores were associated with increased delayed antibiotic prescriptions (χ^2^ = 8.42, *P* = 0.004 for FeverPAIN ≥4; χ^2^ = 32.0, *P*<0.001 for Centor ≥3).

**Conclusion:**

In those who do not require immediate antibiotics in primary care, neither CPR provides a reliable way of diagnosing streptococcal throat infection. However, clinicians were more likely to give delayed prescriptions to those with higher scores.

## How this fits in

Sore throat is one of the most common presentations in primary care, and despite the self-limiting nature, antibiotics are overprescribed more for this than any other condition. There remains ambiguity as to the performance of FeverPAIN and Centor criteria in ruling out streptococcal infection. This diagnostic accuracy study from 565 participants in UK primary care, shows neither CPR performs well in a low-prevalence setting. Clinicians can be reassured that either no or a delayed antibiotic prescription is appropriate for the majority of patients.

## Introduction

Sore throats are a common presentation in UK primary care, accounting for around 3.5 million appointments per year.^1^ The majority of infections are viral, and risk of progression to serious complications is low (approximately 1% of patients).^2^ Despite this, antibiotics are frequently prescribed; roughly 60%–70% of consultations in 2010–2011.^1^ Sore throats are more frequently associated with inappropriate prescriptions than any other condition.^3^

NICE guidelines on sore throat infection recommend use of two CPRs to inform antibiotic prescribing strategies.^4^ The Centor score was developed in the 1980s in adults presenting to emergency departments, and allocates one point to presence of cervical lymph nodes, fever, tonsillar exudates, or absence of cough.^5^ NICE recommends immediate or delayed antibiotics for a score of ≥3, associated with a 32–56% chance of streptococcal infection. The Centor criteria have been criticised for leading to overprescription of antibiotics in primary care settings^6^ and a recent meta-analysis has highlighted its limitations, particularly to ‘rule in’ infection.^7^ It was also developed to detect group A streptococcus (GAS) infection only. FeverPAIN was derived from UK primary care populations and is a 5-point scale with a point for fever, absence of cough, and purulent tonsils, as per Centor, but also for severe tonsillar exudate and duration of symptoms of less than 3 days.^8^ It incorporates three clinical decisions: no antibiotics (0–1); a delayed prescription (2-3);^2,3^ and immediate antibiotics (4-5),^4,5^ with scores in the latter category associated with a 60–65% chance of group A, C, or G streptococcal infection. Despite endorsement from NICE,^4^ it has only been tested in one randomised controlled trial (RCT) outside the original study population.^9^ Thus, there remains uncertainty about both the performance and validity of each CPR in clinical practice.

The TOAST (Treatment Options without Antibiotics for Sore Throat) was a double-blinded RCT, in which adults presenting to their primary care clinician with acute sore throat were randomised to receive a single dose of dexamethasone or placebo. Those who needed immediate antibiotics were not recruited to the trial, but as part of the pragmatic nature, clinicians could opt for a delayed prescription. Throat swabs were taken on initial assessment and symptom diary cards were recorded for the first 7 days. Results of the study have previously been reported,^10^ with steroids making no difference to symptom burden in the first 24 hours. The detailed data collection of sore throat symptomatology, duration, and aetiology makes this a suitable cohort. The validity of each CPR can be examined in a population deemed not to require immediate antibiotics, with a focus on the delayed prescription strategy, given recent evidence that delayed prescriptions may reduce antibiotic prescribing rates without compromising on clinical safety.^11^

The aims of this study were to retrospectively analyse TOAST data to understand:

1. The diagnostic accuracy of Centor and FeverPAIN, compared with microbiological culture of throat swabs, in identifying streptococcal throat infections in a low-risk population.
2. The relationship between CPR and prescription decision in a cohort where this was not explicitly recorded.

## Method

### Participant recruitment and baseline assessment for TOAST

Participants aged ≥18 years were recruited on the day of presentation to their GP practice with acute symptoms of sore throat (onset within the last 7 days) and odynophagia judged by the clinician to be infective in origin. Exclusion criteria included: recent (<1 month) use of inhaled or oral corticosteroids or adenotonsillectomy, recent use (<14 days) of antibiotics, or a clear alternative diagnosis. Before randomisation, the clinician was free to offer either no antibiotics or a delayed antibiotic prescription, typically to take after 48 hours if symptoms had not improved. Participants were randomised and treated immediately with either a single dose of 10 mg oral dexamethasone or matching placebo.

Data required for calculation of FeverPAIN and Centor scores were obtained at baseline, using standardised questionnaires, by trained clinicians, but scores were not documented. A throat swab was taken for microbiological culture.

Participants were asked to complete a symptom diary for 7 days post-randomisation. Sore throat and pain on swallowing were recorded daily on a validated^12^ 7-point Likert scale. Use of over-the-counter medications and antibiotic consumption were also recorded.

### Identification of streptococcal throat infections

Throat swabs were collected at baseline and sent to the central laboratory for culture and sensitivity. A positive swab was reported if any Lancefield beta-haemolytic group A, C, and G streptococcus was isolated, given the overlapping clinical syndromes and sequelae.^13^ Additional analysis was performed focused on solely GAS infections, which are responsible for the majority of infections, and the only strain that is identified on most commercially available rapid antigen testing kits.

### Clinical prediction rules (CPRs)

NICE guidelines use both CPRs to employ thresholds below which no antibiotics are recommended and above which either a delayed or immediate antibiotic prescription should be considered.^4^ The authors chose, as the main comparison, a high probability of streptococcal throat infection for both CPRs, that is, FeverPAIN ≥4 or Centor ≥3, where delayed or immediate antibiotics may be given. A freely available online calculator (Medical Test Calculator) was used to produce an infographic of how diagnostic accuracy results would translate in to test results for 100 patients presenting with sore throat.^14^ These thresholds were compared with the reference standard of microbiological culture of bacterial throat swab. Additionally, each different score on the CPRs was looked at to see how the likelihood of streptococcal infection varied across this range. Finally, the relationship was examined between delayed prescriptions offered, and the CPR scores. It was not known how clinicians were using CPR scores.

### Statistical analysis

All data analysis was done in Stata (version 14). FeverPAIN and Centor scores were calculated based on data provided in patient baseline assessment. Scores at each threshold of the CPR were compared with bacterial culture of throat swab using sensitivity, specificity, and positive and negative predictive values, with 95% CIs. The overall performance of each CPR was also summarised using the area under ROC curve, with a 95% CI. Pearson’s χ^2^ test was used to compare CPR scores with delayed prescription rates, a *P* value less than 0.05 was considered statistically significant.

## Results

### Trial population

The characteristics of the study population have been described previously.^10^ Five hundred and sixty-five participants were randomised, with a median age of 34 years. Four hundred and twenty-five (75.2%) were female, and 434 (76.8%) were employed or in education. A total of 223 participants (39.5%) received a delayed antibiotic prescription. Most participants had low or medium FeverPAIN or Centor scores; the median score was 1 for both CPRs in the cohort.

Throat swab culture results were available for 502 (89%) participants. Eighty-one (16.1%) were positive for streptococcal group A, C, or G infections, and 63 (12.5%) were positive for group A only (Table 1).

**Table 1.** Throat swab culture results

	All beta-Lancefield group streptococci^a^	Group A streptococci	Group C and G streptococci
Negative swab	Positive swab	*P*	Negative swab	Positive swab	*P*	Negative swab	Positive swab	*P*
*n*, %(*n* = 421)	*n*, %(*n* = 81)	*n*, %(*n* = 439)	*n*, %(*n* = 63)	*n*, %(*n* = 484)	*n*, %(*n* = 18)
Centor ≥3(*n* = 74)	55 (13%)	19 (24%)	0.01	59 (13%)	15 (24%)	0.03	77 (16%)	4 (22%)	0.33
FeverPAIN 2–3(*n* = 266)	192 (46%)	48 (60%)	0.02	205 (47%)	35 (56%)	0.16	253 (52%)	13 (72%)	0.03
FeverPAIN ≥4(*n* = 11)	8 (2%)	3 (3.7%)	0.15	9 (2%)	2 (3%)	0.42	10 (2%)	1 (6%)	0.09

a All beta-Lancefield group streptococci to include group A, C, and G streptococci strains isolated on agar plates after 48 hours culture.

### Association between CPR scores and presence of streptococci on throat swab

**Figure 1.**
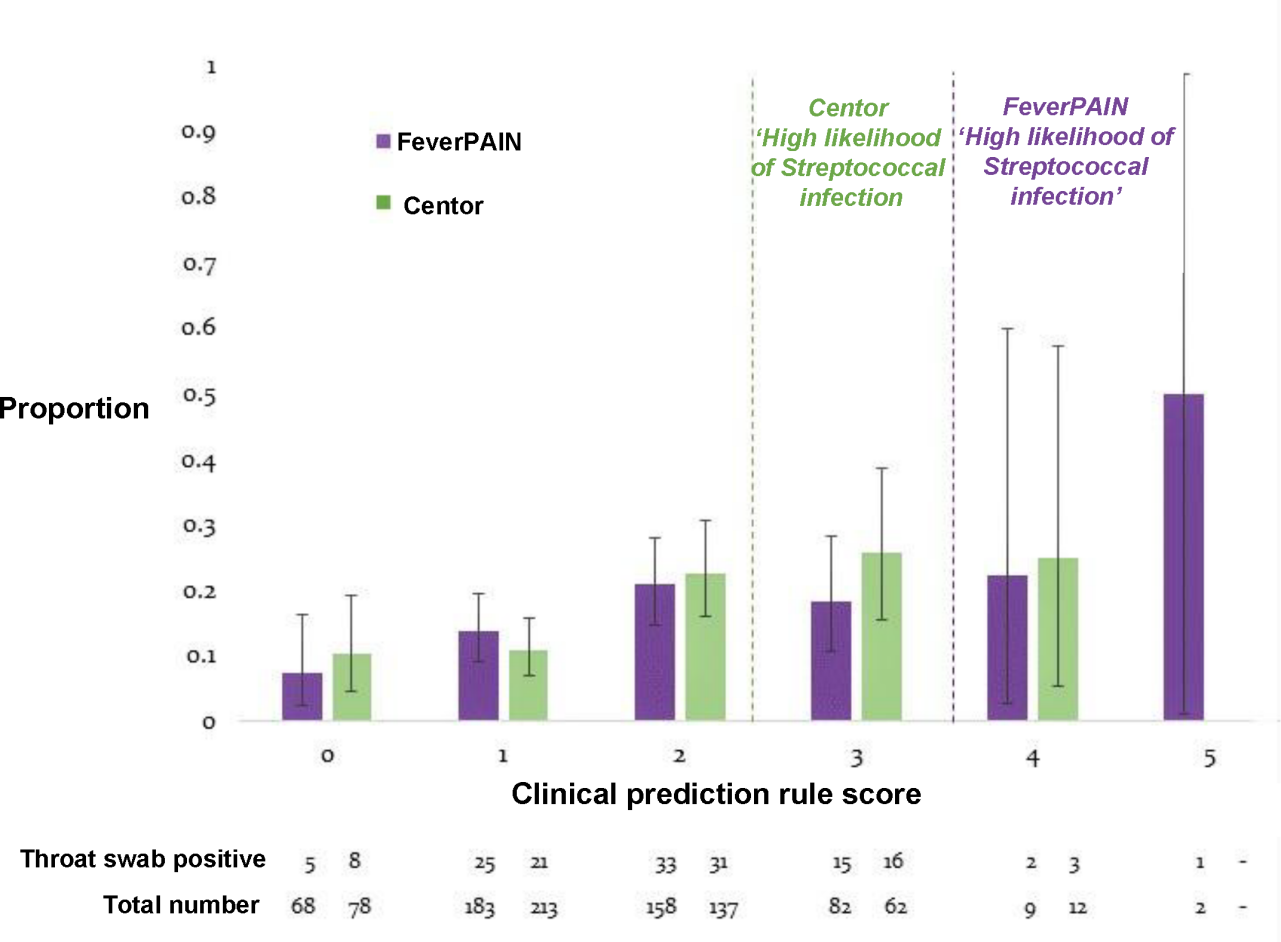
Proportion with throat swabs positive for streptococcal † infection. Proportion of those with streptococcal infection within each clinical prediction score, with 95% confidence intervals represented by black bars. Lines on the graph added for thresholds of Centor criteria ≥3 and FeverPAIN ≥4 indicating high probability of infection, where NICE supports consideration of antibiotic prescription. Data below the graph indicate the number of participants at each score, and number who were throat-swab positive for group A, C, or G streptococci. All beta-Lancefield group streptococci to include group A, C, and G streptococci strains isolated on agar plates after 48 hours culture.

**Figure 2.**
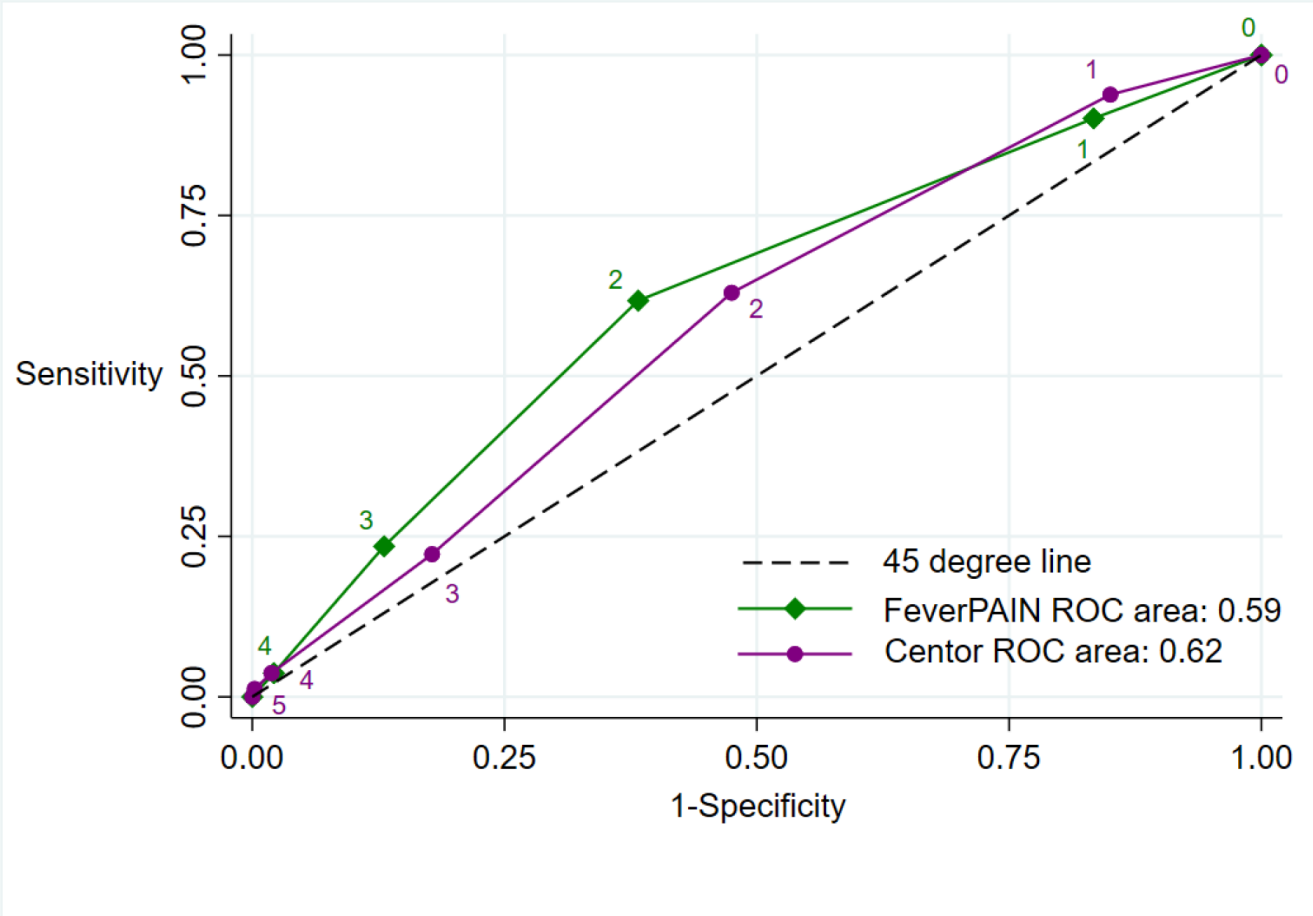
Receiver operating characteristic (ROC) curves for diagnostic accuracy in identifying streptococcal infections for FeverPAIN and Centor clinical prediction rules. All beta-Lancefield group streptococci to include group A, C, and G streptococci strains isolated on agar plates after 48 hours culture. ROC curve analysis showing sensitivity versus 1-specificity each threshold. The different scores are shown next to a point on the graph. Each colour represents the diagnostic accuracy of one clinical prediction rule for streptococcal infection in adults with sore throat.

**Figure 3.**
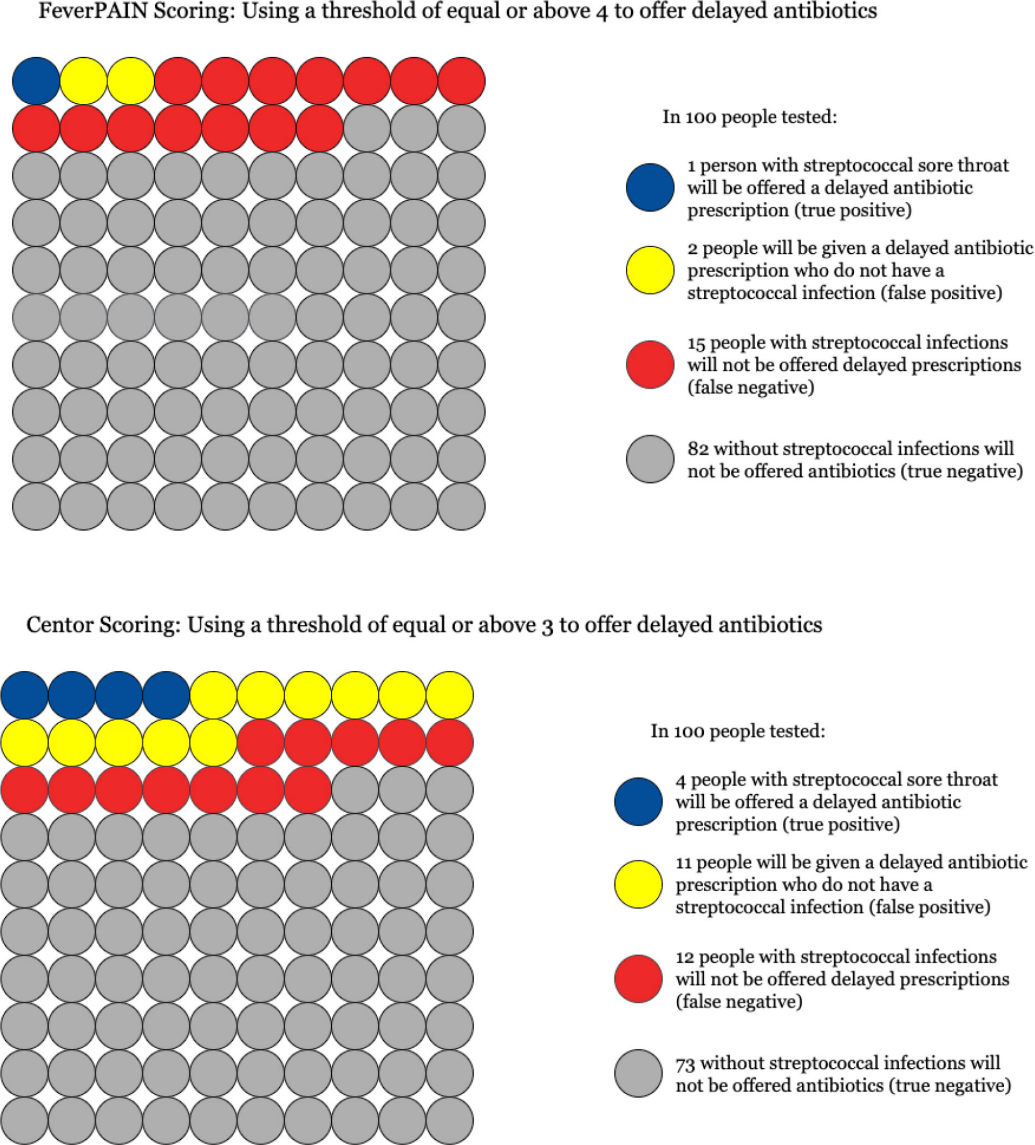
Implications of diagnostic accuracy of FeverPAIN and Centor CPR in diagnosing streptococcal throat infections in 100 adults presenting with sore throat not deemed to require immediate antibiotics. The diagram illustrates how both CPR scores would operate if used on 100 people presenting with sore throat, based on a similar prevalence (16%) of streptococcal infection, as found in the present study. Different colours indicate possible outcomes: true positive (blue), false positive (yellow), false negative (red), and true negative (grey).

Streptococci were detected on throat swabs across the range of scores of both CPRs. Prevalence was low, and positive throat swabs were less common for very low scores (0, or 1) (Figure 1). The overall performances of each CPR were similar and modest. The summary area under ROC curve was 0.62 (95% CI = 0.56 to 0.69) for Centor and 0.59 (95% CI = 0.52 to 0.65) for FeverPAIN (Figure 2). At the aforementioned thresholds for considering antibiotics, sensitivity of both CPRs was low (Table 2). At a FeverPAIN score of ≥4, the specificity was 98.1%, higher than a Centor score of ≥3 (specificity 86.9%). However, the sensitivity of a FeverPAIN score was very low (3.7%). At a prevalence of 16.1%, the post-test probability of a positive test was 25.7% (95% CI = 16.2% to 37.2%) for Centor ≥3% and 27.3% (95% CI = 6.0% to 61.0%) for FeverPAIN ≥4. Figure 3 displays how this may translate into clinical practice. Three in 100 people may have received a delayed prescription for antibiotics using FeverPAIN score of ≥4, with two of these prescriptions being unnecessary. In contrast, 15 people may have received a delayed antibiotic prescription for Centor ≥3, of which 11 would be unnecessary.

**Table 2.** Diagnostic accuracy at each threshold of clinical prediction rule

**Threshold**	***n* above / below**	**Sensitivity** **(95% CI)**	**Specificity** **(95% CI)**	**ROC** **(95% CI)**	**PPV** **(95% CI)**	**NPV** **(95% CI)**
Centor ≥1	424/78	90.1 (81.5 to 95)	16.6 (13.2 to 20.5)	0.53 (0.5 to 0.57)	17.2 (13.7 to 21.2)	89.7 (80.8 to 95.5)
Centor ≥2	211/298	61.7 (50.3 to 72.3)	61.8 (56.9 to 66.4)	0.62 (0.6 to 0.68)	23.7 (18.1 to 30)	89.3 (85.2 to 92.6)
Centor ≥3	74/428	23.5 (14.8 to 34.2)	86.9 (83.3 to 90)	0.55 (0.50 to 0.60)	25.7 (16.2 to 37.2)	85.5 (81.8 to 88.7)
Centor ≥4	12/490	3.7 (0.77 to 10.4)	97.9 (96 to 99)	0.51 (0.49 to 0.53)	25 (5.49 to 57.2)	84.1 (80.5 to 87.2)
FeverPAIN ≥1	434/68	93.8 (86.2 to 98)	15 (11.7 to 18.7)	0.54 (0.51 to 0.58)	17.5 (14.1 to 21.4)	92.6 (83.7 to 97.6)
FeverPAIN ≥2	251/251	63 (51.5 to 73.4)	52.5 (47.6 to 57.4)	0.58 (0.52 to 0.64)	20.3 (15.5 to 25.8)	88 (83.4 to 91.8)
FeverPAIN ≥3	93/409	22.2 (13.7 to 32.8)	82.2 (78.2 to 85.7)	0.52 (0.48 to 0.57)	19.4 (11.9 to 28.9)	84.6 (80.7 to 88)
FeverPAIN ≥4	11/491	3.7 (0.77 to 10.4)	98.1 (96.3 to 99.2)	0.51 (0.49 to 0.53)	27.3 (6.02 to 61)	84.1 (80.6 to 87.2)
FeverPAIN ≥5	2/500	1.29 (0.031 to 6.69)	99.8 (98.7 to 100)	0.51 (0.49 to 0.52)	50 (1.26 to 98.7)	84 (80.5 to 87.1)

ROC = area under receiver operating characteristics curve. PPV = positive predictive value. NPV = negative predictive value

The diagnostic accuracy did not improve for either CPR at different thresholds (Table 2). The negative predictive value (NPV) estimates remained high across all scores, reflective of the prevalence of streptococcal infections in the study population. Using a FeverPAIN score of ≥2 or 'intermediate probability' would have improved the sensitivity to 63% (95% CI = 51.5 to 73.4) but decreased the specificity to 52.5% (95% CI = 47.6 to 57.4). In clinical terms this translates into prescribing 50 in 100 people delayed antibiotics, only 10 of whom have streptococcal infection, and six infections in the remaining 50 people not identified.

### Group A streptococcal infections

Focusing on only GAS infection diminished the diagnostic accuracy of FeverPAIN CPR (Supplementary Figure 1). Results largely remained the same for Centor criteria, and area under summary ROC curve between two CPRs significantly differed (χ^2^ = 4.27, *P* = 0.039). The corresponding positive predictive value (PPV) for a FeverPAIN score ≥4 was 18.2% (95% CI = 2.28% to 51.8%) compared with 20.3% (95% CI = 11.8% to 31.2%) for Centor ≥3.

### Association between scores and likelihood of a delayed prescribing decision

**Figure 4.**
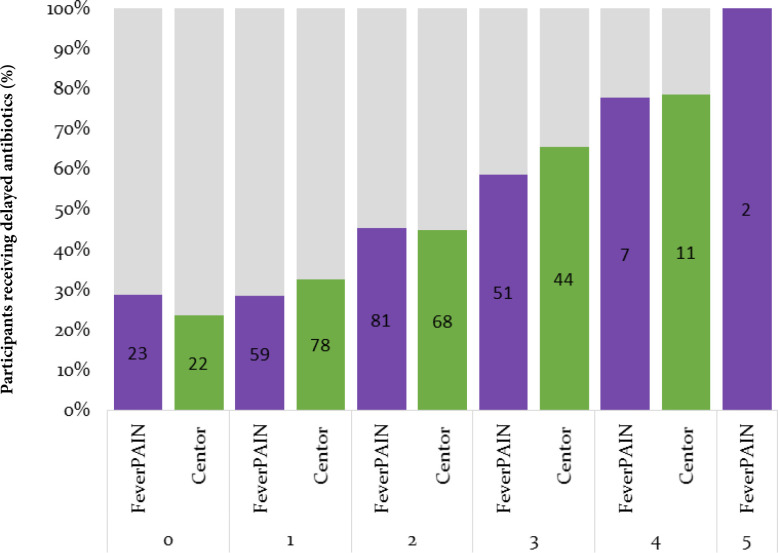
Delayed antibiotics prescribed in TOAST according to each clinical preduction rule score. The graph displays the percentage of participants in each clinical decision score who were prescribed delayed antibiotics at presentation. The numbers within each bar indicate the number of participants who received a delayed prescription at each score.

Overall, 223 (39.1 %) of participants received a prescription for delayed antibiotics. Figure 4 shows a clear relationship between increasing CPR score and the proportion of patients receiving antibiotics. A score at or above the NICE threshold for considering antibiotics was significantly associated with receiving a delayed prescription: χ^2^ = 32.0, *P*<0.001 for Centor scores ≥3 and χ^2^ = 8.42, *P* = 0.004 for FeverPAIN ≥4. Very few of the patients had maximal CPR scores (less than 2% had a FeverPAIN score of ≥4 and 2.5% had a Centor score of 4), suggesting these patients may have received immediate antibiotics, thus excluded from entering the trial. However, not all antibiotic prescribing was so clearly following the NICE guidance. Delayed prescriptions were frequently issued to those with low scores: 29% of those with a FeverPAIN score of 0, and 24% with a Centor score of 0.

## Discussion

### Summary

In patients presenting to primary care who are not deemed to require immediate antibiotics, the results suggest that the two CPRs recommended by NICE have a limited role in deciding who has a streptococcal throat infection.

Overall, the two CPRs were equivalent, both demonstrating poor ability to discriminate between the presence and absence of streptococci, and at lower than previous estimates. For the NICE chosen thresholds for consideration of immediate or delayed antibiotics, a FeverPAIN score of ≥4 had a greater specificity but poorer sensitivity than Centor ≥3. The post-test probability of a positive swab was 6.02% to 61%, for FeverPAIN scores ≥4; a much greater range of uncertainty than 62% to 65% predicted by NICE. Similarly, the PPV for Centor scores of ≥3 were 16.2% to 37.2% compared with the 32% to 56% expected. Given that this is a low-risk population, where the clinician has already decided immediate antibiotics are not needed, using the FeverPAIN score would select a much smaller proportion of patients for potential offer of prescription: *n* = 11/522 (2%) compared with Centor *n* = 74/522 (14%).

While it was not known if, or how, clinicians were formally using either CPR, there was some concordance with clinical decision making, as participants with higher FeverPAIN or Centor scores were more likely to be given delayed antibiotics. However, there was heterogeneity in practice, as antibiotics were prescribed across the range of CPRs, without a clear stepwise change at a single threshold. Overall, many more patients were given delayed prescriptions for antibiotics than either had streptococcal throat infections, or indicated by high probability CPR scores.

### Strengths and limitations

The main strength of the study is that it had detailed data on patient characteristics and microbiological diagnosis through throat swabs for patients presenting to primary care with sore throat. The study was not focused on, and did not require calculation of, a CPR which may have improved the ability to capture ‘natural’ behaviour of clinicians. The study had laboratory data on all three common isolates of streptococcus, rather than only group A strains.

There are limitations to the findings. The study had a similar number of participants to the original FeverPAIN trial, but this was a secondary data analysis, not specifically powered to validate CPRs. Eleven per cent of culture results were missing, thought to be lost, at random, in transit to laboratory by postal services, but this may have altered the results. There may have been some differences in the study population by virtue of the fact that they were recruited into a trial of oral steroids. The spectrum of disease severity in TOAST was low, owing to the inclusion criteria, only 2% of patients had a high probability FeverPAIN score, compared with 8.4% in original derivation study.^2^ In other cohorts, 96.9% of participants with FeverPAIN score 4–5 were given immediate antibiotics.^15^ The diagnostic accuracy of both CPRs is likely to be better in a broader spectrum of patients.^7,13^ Clinicians may also have been more likely to prescribe delayed antibiotics, knowing half of participants would receive immunosuppressant medication.^16^ The chosen reference standard of microbiological throat swab culture is a widely accepted method of identifying streptococcal throat infections, but cannot differentiate between active disease and asymptomatic carriage.^17^

### Comparison with existing literature

The summary ROC for both Centor and FeverPAIN was lower than in the original conception studies.^5,13^ A recent meta-analysis of Centor scoring, across a range of clinical settings, also found a low summary area under ROC curve (0.69) and poor calibration.^7^ The sensitivity of a Centor score of 0 was 96.4% to 97.8%, higher than in the present study's population (81.5% to 95%), suggesting greater confidence to rule out bacterial infection. But above this, the PPV remained low, encouraging the overprescription of antibiotics in most primary care settings. FeverPAIN, during development in the PRImary care Streptococcal Management (PRISM) studies had better diagnostic accuracy compared with the present study, with summary area under ROC curve across two cohorts 0.71 (0.661 to 0.758) and 0.735 (0.69 to 0.78).^9^ Better performance in PRISM may be owing to differences in the trial populations; prevalence of streptococci in PRISM was 34%, as those who required immediate antibiotics were not excluded, compared with 16% in TOAST.^13^ Children aged >5 years, who may have different features of symptomatic streptococcal throat infection^18^ and have greater asymptomatic carriage,^17,19^ were also included. The finding that a high proportion of those at low risk still receive the offer for antibiotics has been reported elsewhere.^15^

### Implications for research and practice

The prevalence of streptococci infections was low. Coupled with the low rate of serious complications of upper respiratory tract infections in UK primary care, this should help reinforce confidence in the clinical decision that when immediate antibiotics are not deemed necessary, they are unlikely to be required. This may be applicable not just to practitioners in GP surgeries, but also other primary care providers expected to see patients with low severity symptoms; for example, pharmacies or minor illness centres. Future research should focus on deprescribing strategies. This may include rapid antigen testing for high CPR scores, as recently piloted in Wales,^20^ or CPR validation studies in broader populations.
